# Expression of CD68 positive macrophages in the use of different barrier materials to prevent peritoneal adhesions—an animal study

**DOI:** 10.1007/s10856-016-5821-3

**Published:** 2016-12-19

**Authors:** Christoph Brochhausen, Volker H. Schmitt, Andreas Mamilos, Christine Schmitt, Constanze N.E. Planck, Taufiek K. Rajab, Helmut Hierlemann, C. James Kirkpatrick

**Affiliations:** 1 REPAIR-lab, Institute of Pathology, University of Regensburg, Regensburg, Germany; 2 Cardiology I, Centre for Cardiology, University Medical Centre, Johannes Gutenberg University of Mainz, Mainz, Germany; 3 Department of Internal Medicine, St. Vincenz and Elisabeth Hospital of Mainz (KKM), Mainz, Germany; 4 Department of Gynaecology and Obstetrics, University of Tuebingen, Tuebingen, Germany; 5 German Centre of Biomaterials and Artificial Organs e.V. Denkendorf, Denkendorf, Germany; 6 Brigham and Women’s Hospital, Harvard Medical School, Boston, USA; 7 Institute of Textile Technology and Process Engineering, Denkendorf, Germany; 8 Department of Biomaterials, Sahlgrenska Academy, University of Gothenburg, Gothenburg, Sweden

## Abstract

In preventing postoperative adhesion formation the optimal barrier material has still not been found. It is therefore imperative to assess the biocompatibility of potential barrier devices. Macrophages play a decisive role in the regulation of wound healing, tissue regeneration and foreign body reaction. Since the number of CD68-positive macrophages represents an important parameter within biomaterial testing, in the present study it was analysed whether a correlation exists between the total number of CD68-positive macrophages and the extent of fibrosis or inflammation in peritoneal adhesion prevention using biomaterials. After standardized peritoneal wounding, Wistar rats were treated with five adhesion barriers or remained untreated as a control. After 14 days, animals were sacrificed and the treated areas were evaluated histomorphologically and immunohistologically. A heterogeneous pattern of macrophage count in relation to fibrosis or inflammation was found. While some groups described a moderate macrophage infiltration without fibrosis, others showed similar numbers of macrophages, but accompanied by moderate fibrosis. Moreover, a minimal number of macrophages was associated with minimal fibrosis. Mild inflammation was seen both with minimal and moderate macrophage infiltration. Altogether, no correlation could be established between the tissue response and the count of CD68-positive macrophages. With a view to macrophage heterogeneity further studies are required to determine the different macrophage subpopulations and clarify the role of these in the tissue responses to barrier materials.

## Introduction

Postoperative adhesion formation occurs in up to 90% of patients undergoing abdominal or pelvic surgery [1] and remains a clinical burden within all surgical specialties [2]. Peritoneal adhesions cause serious symptoms like infertility, which often affects young women [3], as well as intestinal obstruction [4, 5], chronic abdominal and pelvic pain [6–8]. Furthermore, the treatment of peritoneal adhesion formation causes costs up to US $1.3 billion per year in the United States alone [9, 10].

As a result of a complex cellular cascade, postoperative adhesion formation is not yet completely understood. Various humoral factors such as cytokines and signalling molecules are crucially involved. The intact peritoneum, consisting of highly functionalized mesothelial cells, creates smoothness of all surfaces inside the peritoneal cavity and ensures frictionless gliding of intraperitoneal organs by producing surfactant [11, 12] and phosphatidylcholine [13]. In addition, mesothelial cells also have antithrombogenic properties and express tissue plasminogen activator (tPA) as well as plasminogen activator inhibitor (PAI) to provide the fibrinogenic and fibrinolytic balance inside the peritoneal cavity [14]. Furthermore, the mesothelium is actively involved in the regulation of the inflammatory response and the coagulation system by expressing cell adhesion molecules [15–18]. During peritoneal damage the functionality of mesothelial cells is disturbed [16]. As a result, tissue damage is followed by local ischaemia, inflammation and an imbalance of the procoagulatory-fibrinolytic system with a predominance of procoagulation [14]. Pathophysiologically, fibrin is exudated as one of the very first steps during the wound healing process after tissue damage. In adhesion formation the preference for coagulation and the inhibition of the fibrinolytic system lead to a decreased fibrin degradation compared to normal conditions [19]. Subsequently, the formation of fibrin bridges to neighbouring tissue occurs and these become organized into a connective tissue, which is the final stage of adhesion formation [20–23]. Clinically, the main strategy in the prevention of postoperative adhesion formation is the use of physical barriers, which separate wounded areas from their environment and so prevent the formation of fibrin bridges and hence peritoneal adhesions [24]. Various barrier materials are available in the form of solid or viscous biomaterials and are currently in clinical use (Table 1) [25, 26].

**Table 1:** The barrier materials used in this study

Barrier	Manufacturer	Components
Adept^®^	ML Laboratories PLC, Hampshire, UK	Icodextrin, sodium chloride, sodium lactate, calcium chloride, magnesium chloride
Spraygel^®^	Confluent Surgical Inc.,Waltham, MA, USA	Polyethylene-glycol, water
Intercoat^®^	Ethicon, Somerville, NJ, USA	Carboxymethylcellulose, polyethylene oxide
Seprafilm^®^	Genzyme, Cambridge, MA, USA	Sodium hyaluronic acid with carboxymethylcellulose
SupraSeal^®^	PolyMedics Innovations GmbH,	Poly-DL-lactic acid, trimethylencarbonate Denkendorf, Germany and ε-caprolactone

Macrophages play a decisive role in inflammation and in wound healing [27]. These cells are able to produce proinflammatory cytokines and to phagocytose in order to eliminate pathogens as well as foreign materials [28]. Furthermore, macrophages secrete various growth factors and signalling molecules and are thus involved in the regulation of inflammation, wound healing and tissue repair [29]. Since these processes are part of adhesion formation, macrophages are crucially involved in the regulation and modulation of their formation and in the tissue reaction to different barrier materials. To gain further insight into the role of macrophages in the tissue response to barrier materials used to prevent the formation of postoperative adhesions, the present study evaluated the macrophage response semi-quantitatively after the application of five different adhesion barrier materials in an animal model. These were Adept^®^, Intercoat^®^, Spraygel^®^, Seprafilm^®^, SupraSeal^®^ and one control group. Since the count of macrophages is an important parameter in biocompatibility testing according to the ISO standard 10993-6 “Biological evaluation of medical devices—Part 6: Tests for local effects after implantation” [30], no attempt was made to identify macrophage subpopulations, but rather to delineate whether the total macrophage infiltration correlates with the tissue response as seen by total inflammation and fibrosis.

## Materials and methods

### The animal study

This study was approved by The Ethics Committee of the Institutional Review Board, University of Tuebingen, Tuebingen, Germany (trial number F1-06).

The female Wistar rats (Charles River, Sulzfeld, Germany) used in this investigation had a weight range between 230–270 g and were housed under standardised laboratory conditions (temperature 21 ± 2 °C, humidity 55% ± 10%, 12:12 h light-dark-cycle). All interventions were performed by the same surgeon and by using powder-free gloves under aseptic conditions. The animals were anaesthetised with ketamine (100 mg/kg) and xylacine (5 mg/kg).

### Experimental adhesion induction

After a midline incision of 5 cm length and opening of the peritoneal cavity, bilateral peritoneal damage of a dimension of 20 × 5 mm was induced via electrocautery (Vio 300D bipolar generator set to 40 W; ERBE Elektromedizin, Tubingen, Germany), and subsequently five interrupted sutures were placed (3–0 Vicryl; Ethicon, Somerville, NJ). Afterwards, the lesions were treated with one of the following commercially available barrier materials: Adept^®^ (Baxter, Deerfield, Illinois, USA) (*n* = 7), Intercoat^®^ (Ethicon, New Brunswick, New Jersey, USA) (*n* = 7), Spraygel^®^ (Confluent Surgical Inc., Waltham, Massachusetts, USA) (*n* = 7), Seprafilm^®^ (Genzyme, Cambridge, Massachusetts, USA) (*n* = 7) or SupraSeal^®^ (PolyMedics Innovations GmbH, Denkendorf, Germany) (*n* = 7). The used barriers in this study and their components are summarized in Table 1. As control, one group remained untreated after laparotomy and peritoneal damage. Each group contained seven animals. The midline incision was then closed and the animals were treated with subcutaneous buprenorphine (0.05 mg/kg) for analgesia. Fourteen days postoperatively, the animals were sacrificed with CO_2_ and the treated tissue was explanted and fixed in buffered formalin (4%).

### Tissue preparation and histological staining

The fixed tissue was dehydrated via standardised, automated protocols and embedded in paraffin. From the paraffin blocks sections of 4 µm thickness were cut. For histological evaluation, the specimens were stained with haematoxylin-eosin (HE) to evaluate the overall histology of the tissue and chloracetate esterase (ASD) to evaluate the amount of granulocytes. Furthermore, the Elastica van Gieson (EvG) stain was performed to score the extent of fibrosis. All histochemical staining reactions were performed according to standardized operating procedures.

For the quantification of macrophages immunohistochemical staining of CD68 was performed using a monoclonal antibody (dilution 1:600, Santa Cruz Biotechnology, Heidelberg Germany) with help of a standardized automated immunostaining-kit (Dako Cytomation Autostainer Plus, Dako, Hamburg, Germany). The nuclei were counterstained with haematoxylin (EN Vision^TM^ Flex Haematoxyline, Dako Denmark A/S, Glostrup, Denmark).

### Data acquisition and evaluation

The evaluation occurred semi-quantitatively. The inflammatory response was investigated in all specimens by determining the population of polymorphonuclear granulocytes in the ASD-staining and of lymphocytes/plasma cells in the HE stain. Furthermore, the foreign body-type multinucleated giant cells were counted in the HE stain. The extent of fibrosis was evaluated in the Elastica van Gieson staining. Macrophages were counted with the help of CD68 immunostaining. All specimens were analysed for each stain and for every variable by evaluating a total of 20 high power fields (magnitude ×400) using an Olympus BX40 light microscope (Olympus GmbH, Hamburg, Germany). In each specimen, ten of these high power fields were randomly chosen and scored within the lesion. For this purpose, high power fields were investigated which were near the barrier material. In the control group and in the case no material was histologically detected the high power fields were chosen near the wound or peritoneal adhesion respectively. The other ten high power fields assessed the tissue at a distance from the treated area. The cells of interest were counted and the amount of fibrosis was scored likewise adjacent to and at a distance from the lesion. In the evaluation the values of cell count and fibrosis in the non-treated region were subtracted from the values within the treated area. This generated a so called ‘barrier value’, by which the pathophysiological conditions other than the treatment were eliminated from the score. The generation of the ‘barrier value´ enabled the evaluation and comparison of the isolated effect of the barriers onto the tissue. Consequently, in this model, the tissue response to the specific barrier material can be considered objectively by minimizing the potential effects of the individual animal’s basal reaction to the wound healing process itself.

### Evaluation score

The evaluation score of the “ISO 10993-6: Biological evaluation of medical devices—Part 6: Tests for local effects after implantation” was chosen to assess the histological findings (Table 2) [30].

**Table 2:** The evaluation score of the tissue response according to [30]

Tissue response	Score				
	None	Minimal	Mild	Moderate	Severe
Polymorphonuclear granulocytes	0	Rare, 1–5/hpf*	5–10/hpf	Heavy infiltrate	Packed
Lymphocytes/plasma cells	0	Rare, 1–5/hpf	5–10/hpf	Heavy infiltrate	Packed
Foreign body-type multinucleated giant cells	0	Rare, 1–2/hpf	3–5/hpf	Heavy infiltrate	Sheets
CD68-positive macrophages	0	Rare, 1–5/hpf	5–10/hpf	Heavy infiltrate	Packed
Fibrosis	0	Narrow band	Moderate band	Broad band	Extensive band

* *hpf* high power field (400× magnitude)

### Statistical analysis

**Fig. 1:**
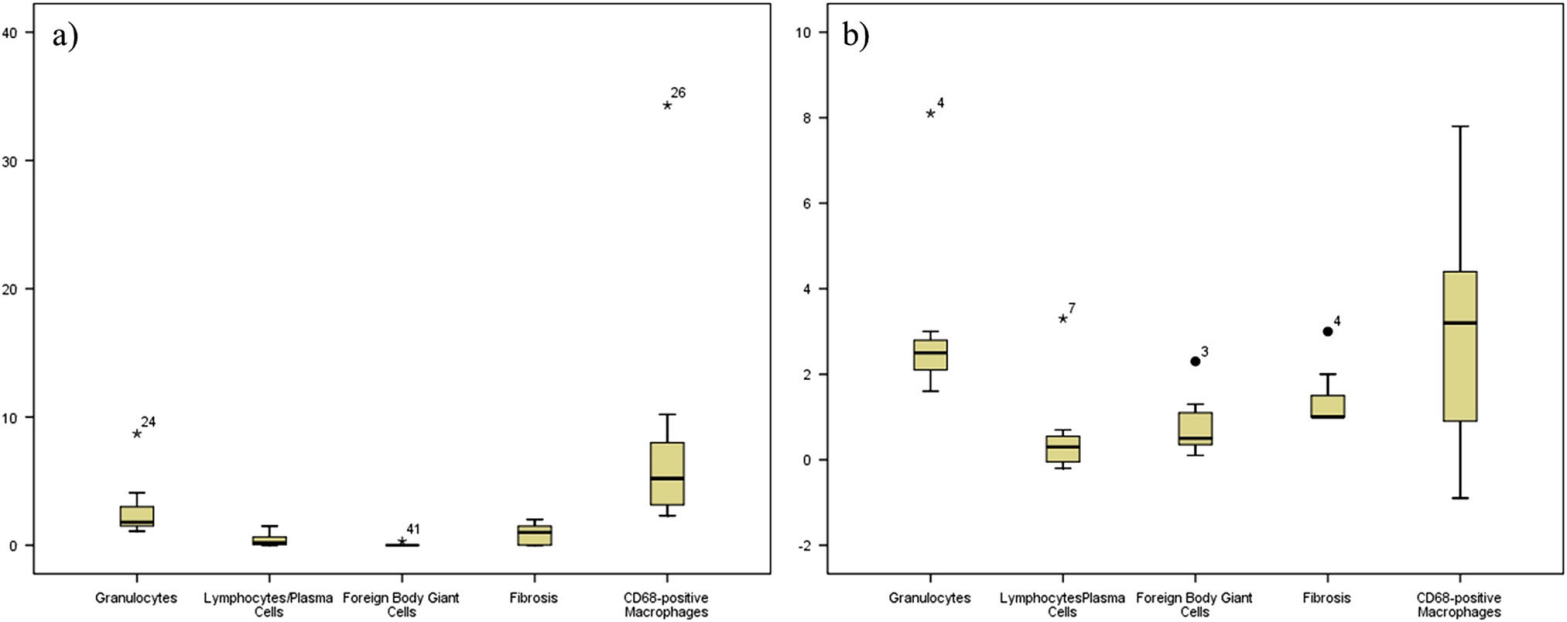
Comparison of the tissue response of the (**a**) control group and (**b**) animals treated with Adept^®^ regarding the count of cells and degree of fibrosis using boxplots. Minimal and mild macrophage infiltrations were both accompanied with a minimal inflammatory response in these groups. Also, in these animals minimal fibrosis was accompanied with mild (control group) and minimal (Adept^®^) infiltration of macrophages

**Fig. 2:**
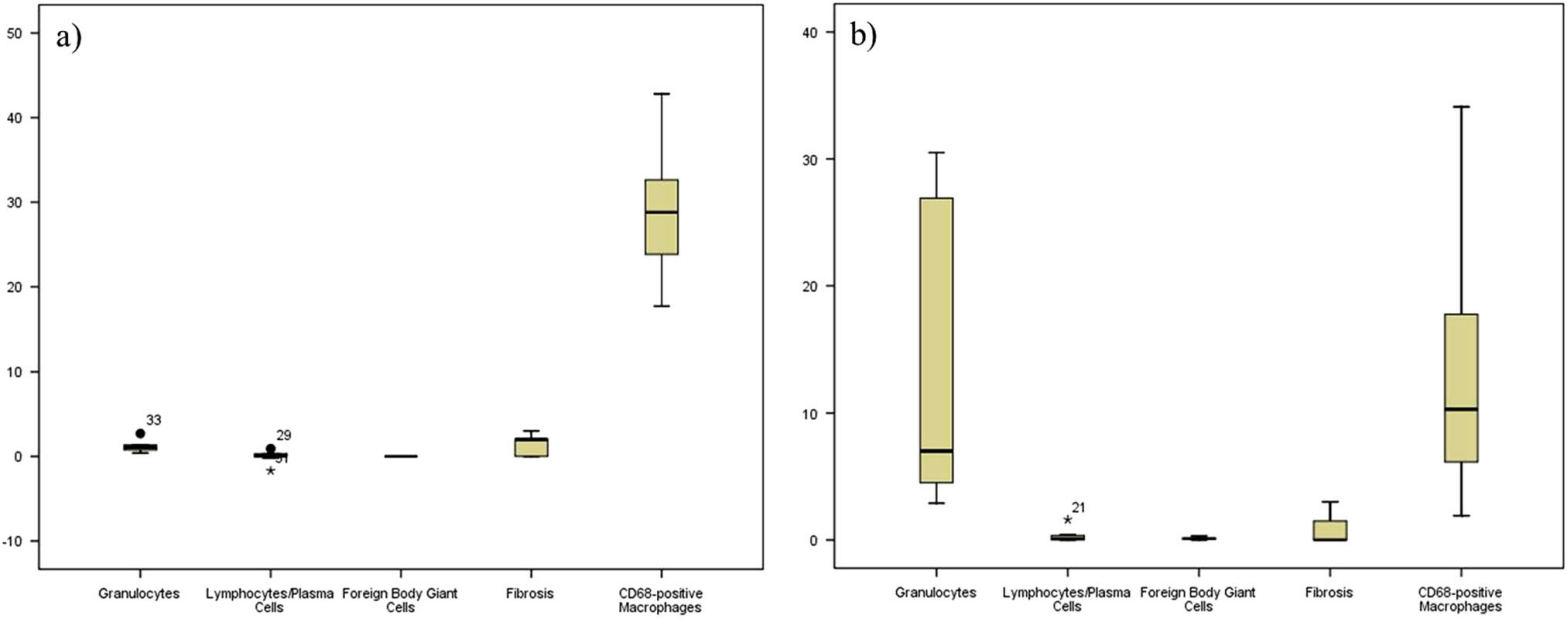
The tissue response to (**a**) the Intercoat^®^ and (**b**) the Spraygel^®^ group regarding polymorphonuclear granulocytes, lymphocytes/plasma cells, foreign body giant cells, fibrosis and macrophages. Here, moderate macrophage infiltration was accompanied with a minimal inflammatory response in the animals treated with Intercoat^®^ and mild inflammation in the Spraygel^®^ group. Regarding fibrosis, both a moderate extent (Intercoat^®^) and no fibrosis (Spraygel^®^) were seen with moderate macrophage infiltration

**Fig. 3:**
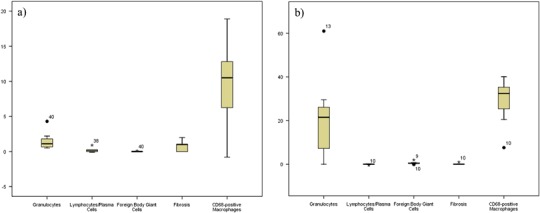
Boxplots showing the tissue response to (**a**) Seprafilm^®^ and (**b**) SupraSeal^®^. In these groups moderate macrophage infiltration was seen with both a minimal (Seprafilm^®^) and a moderate inflammatory response (SupraSeal^®^). No fibrosis was seen with moderate macrophage infiltration in the animals treated with SupraSeal^®^ whereas in the Seprafilm^®^ group minimal fibrosis was accompanied with a moderate infiltration of macrophages. Taken the results of Figs. 1–3 together, the count of macrophages did not go along with any type of tissue response

SPSS Statistics 17.0 was used for statistical analyses. All groups were compared by pairs with the Mann-Whitney-U test and the Kruskal-Wallis test at a significance level of *p* < 0.05. Regarding the extent of fibrosis, the chi-square test and contingency tables were used. The statistical results (median, first quarter (Q1), third quarter (Q3), minimum, maximum) are given in Tables 3–5 and are illustrated in Figs. 1–3 via boxplots. In the following text, the median is described for each variable in each group. Further, Pearson product-moment correlation coefficient was used for correlation analysis.

**Table 3:** Semi-quantitative results of the control- and the Adept^®^ group

Barrier	Tissue response		Median	Q1/Q3	Minimum	Maximum
Control group	Granulocytes	Near the lesion	3.5	2.4/5.1	2.1	12.0
		Afar from the lesion	1.4	0.6/2.4	0.4	3.3
		Barrier value	1.8	1.3/4.1	1.1	8.7
	Lymphocytes/plasma cells	Near the lesion	0.2	0/1.1	0	1.6
		Afar from the lesion	0	0/0.1	0	0.1
		Barrier value	0.2	0/1	0	8.7
	Macrophages	Near the lesion	7.1	5.2/10.2	2.9	36.7
		Afar from the lesion	1.9	0.6/2.4	0	2.7
		Barrier value	5.2	3/10.2	2.3	34.3
	Foreign body-type multinucleated	Near the lesion	0	0/0	0	0.3
	giant cells	Afar from the lesion	0	0/0	0	0
		Barrier value	0	0/0	0	0.3
	Band of fibrosis	Near the lesion	Moderate	Moderate/broad	Narrow	Broad
		Afar from the lesion	Narrow	Narrow/moderate	Narrow	Moderate
		Barrier value	Narrow	None/moderate	None	Moderate
Adept^®^ group	Granulocytes	Near the lesion	4	3.8/6.7	3.7	8.7
		Afar from the lesion	1.7	1.3/2.4	0.6	3.7
		Barrier value	2.5	1.8/3	1.6	8.1
	Lymphocytes/plasma cells	Near the lesion	0.4	0.1/0.7	0	3.3
		Afar from the lesion	0.1	0/0.2	0	0.3
		Barrier value	0.3	−0.1/0.7	−0.2	3.3
	Macrophages	Near the lesion	5.4	1.3/7.4	0.5	12.8
		Afar from the lesion	1.4	0.8/4.5	0.5	5
		Barrier value	3.2	0.8/4.6	−0.9	7.8
	Foreign body-type multinucleated	Near the lesion	0.6	0.3/1.4	0.2	2.4
	giant cells	Afar from the lesion	0.1	0/0.1	0	0.1
		Barrier value	0.5	0.3/1.3	0.1	2.3
	Band of fibrosis	Near the lesion	Moderate	Moderate/broad	Moderate	Extensive
		Afar from the lesion	Narrow	Narrow/narrow	Narrow	Moderate
		Barrier value	Narrow	Narrow/moderate	Narrow	Broad

**Table 4:** Semi-quantitative results of the Intercoat-^®^ and the Spraygel^®^ group

Barrier	Tissue response		Median	Q1/Q3	Minimum	Maximum
Intercoat^®^ group	Granulocytes	Near the lesion	1.4	1/2.6	0.5	3.2
		Afar from the lesion	0.4	0.2/0.5	0.1	1.2
		Barrier value	1.1	0.6/1.4	0.4	2.7
	Lymphocytes/plasma cells	Near the lesion	0.3	0.2/1	0.2	1.6
		Afar from the lesion	0.1	0.1/0.7	0	2.7
		Barrier value	0.1	−0.2/0.3	−1.7	0.9
	Macrophages	Near the lesion	44.9	28/57.5	21.7	80.8
		Afar from the lesion	12.2	1.2/28.1	0.8	50.7
		Barrier value	28.8	20.5/35.2	17.7	42.8
	Foreign body-type multinucleated	Near the lesion	0	0/0	0	0
	giant cells	Afar from the lesion	0	0/0	0	0
		Barrier value	0	0/0	0	0
	Band of fibrosis	Near the lesion	Broad	Narrow/extensive	Narrow	Extensive
		Afar from the lesion	Narrow	Narrow/narrow	Narrow	Moderate
		Barrier value	Moderate	None/moderate	None	Broad
Spraygel^®^ group	Granulocytes	Near the lesion	9.1	4.6/29.4	4.4	32.2
		Afar from the lesion	1.7	0.8/2.1	0.5	3.1
		Barrier value	7	3.9/28.6	2.9	30.5
	Lymphocytes/plasma cells	Near the lesion	0.2	0/0.4	0	1.7
		Afar from the lesion	0	0/0.1	0	0.1
		Barrier value	0.1	0/0.4	0	1.6
	Macrophages	Near the lesion	24.2	7.4/29.8	6.8	41.6
		Afar from the lesion	7.5	2.5/9.3	1.1	18.3
		Barrier value	10.3	4.9/20.5	1.9	34.1
	Foreign body-type multinucleated	Near the lesion	0.1	0/0.2	0	0.3
	giant cells	Afar from the lesion	0	0/0	0	0
		Barrier value	0.1	0/0.2	0	0.3
	Band of fibrosis	Near the lesion	Moderate	Narrow/broad	Narrow	Extensive
		Afar from the lesion	Narrow	Narrow/moderate	Narrow	Moderate
		Barrier value	None	None/moderate	None	Broad

**Table 5:** Semi-quantitative results of the Seprafilm-^®^ and the SupraSeal^®^ group

Barrier	Tissue response		Median	Q1**/**Q3	Minimum	Maximum
Seprafilm^®^ group	Granulocytes	Near the lesion	1.6	1/3.9	0.9	5.1
		Afar from the lesion	0.5	0.4/0.8	0.3	2.5
		Barrier value	1.1	0.5/2.2	0.5	4.3
	Lymphocytes/plasma cells	Near the lesion	0.2	0.1/0.2	0	1.3
		Afar from the lesion	0	0/0.3	0	0.4
		Barrier value	0.2	0/0.2	−0.1	0.9
	Macrophages	Near the lesion	23.8	13.1/24.8	1	48.1
		Afar from the lesion	7	2.6/19.6	1.8	33.6
		Barrier value	10.5	5.2/14.5	−0.8	18.9
	Foreign body-type multinucleated	Near the lesion	0	0/0	0	0.1
	giant cells	Afar from the lesion	0	0/0	0	0
		Barrier value	0	0/0	0	0.1
	Band of fibrosis	Near the lesion	Moderate	Narrow/broad	Narrow	Broad
		Afar from the lesion	Narrow	Narrow/narrow	Narrow	Broad
		Barrier value	Narrow	None/narrow	None	Moderate
SupraSeal^®^ group	Granulocytes	Near the lesion	23.2	3.8/30.1	0.6	62.9
		Afar from the lesion	0.7	0.6/1.7	0.4	1.9
		Barrier value	21.5	2.7/29.5	0	61
	Lymphocytes/plasma cells	Near the lesion	0	0/0.2	0	0.4
		Afar from the lesion	0	0/0.1	0	0.9
		Barrier value	0	0/0.1	−0.5	0.1
	Macrophages	Near the lesion	36.6	22.5/47.2	11.9	54.3
		Afar from the lesion	4.3	2/14.2	1.3	16.9
		Barrier value	32.4	20.5/37.4	7.6	40.1
	Foreign body-type multinucleated	Near the lesion	0.5	0.3/0.8	0	2.1
	giant cells	Afar from the lesion	0	0/0.1	0	0.2
		Barrier value	0.5	0.3/0.7	−0.1	2
	Band of Fibrosis	Near the lesion	Narrow	Narrow/narrow	Narrow	Broad
		Afar from the lesion	Narrow	Narrow/narrow	Narrow	Moderate
		Barrier value	None	None/none	None	Narrow

## Results

### Overall tissue response

First, the tissue response to each barrier is described to outline the impact of each material on the tissue (Figs. 1–3, Tables 3–5).

The time-point 14 days was used for the histo-morphological analysis, since at that time the material-induced macrophage-flux could be analysed. During normal peritoneal wound healing the maximum of macrophage infiltration is given by the days 2–4, and the serosal wound healing is always completed within day 10–11 [22]. In this scenario fibroblast infiltration and collagen formation is also completed after day 10–11. So we assumed the reaction that was observed at day 14 was given due to the cellular reaction on the used biomaterial.

The definition of a fibrosis was given according to the ISO-standard by an increase of collagen fibres and fibroblasts resulting in a dense cellular intermingled collagen network, which properly could be illustrated within the Elastica van Gieson staining [26]. The thickness of these bands was defined in “narrow band,” “moderate band,” “broad band” and “extensive band” [29].

#### The control group

**Fig. 4:**
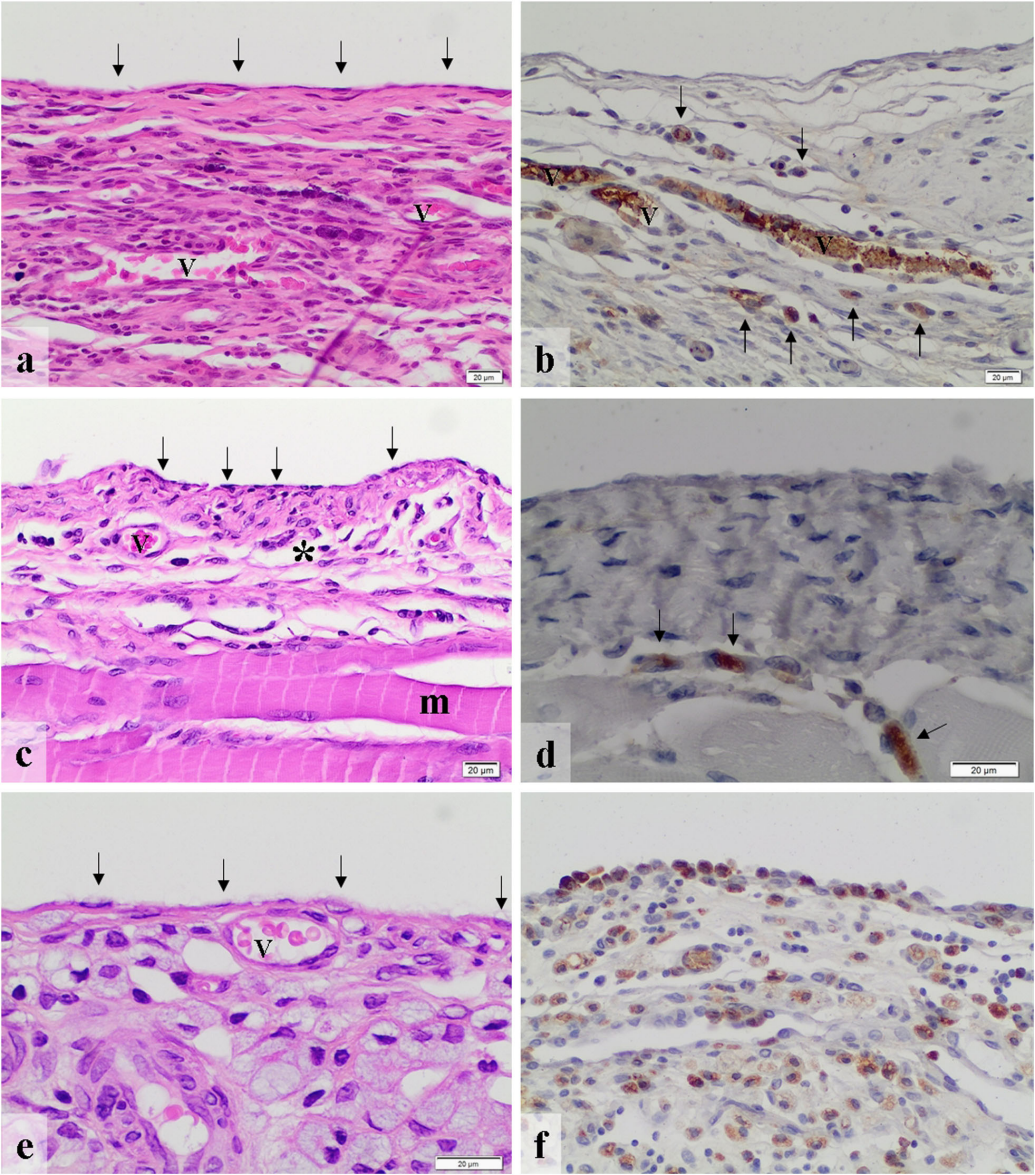
(**a**) The control group shows a flat mesothelial cell layer (*arrows*) and blood vessels (v) (HE ×200) as well as (**b**) some macrophages (arrows) (CD68-staining ×200). (**c**) In the Adept^®^ group, flat mesothelium (*arrows*)-covered connective tissue (*) with blood vessels (v) and subjacent muscle (m) are seen (HE ×200). (**d**) Some macrophages (arrows) were observed within the submesothelium (CD68 staining ×400). (**e**) Intercoat^®^ revealed flat mesothelial cells (*arrows*) on top of connective tissue with blood vessels (v) (HE ×400) and (**f**) a considerable infiltration with (*arrows*) macrophages (CD68 staining ×200)

The animals of the control group (*n* = 7) (Table 3) showed a flat mesothelial cell layer and blood vessels with only minimal infiltration with polymorphonuclear granulocytes and no lymphocytes or plasma cells in the areas near the lesion (Fig. 4a). There was a mild infiltration with macrophages (Fig. 4b), but no foreign body-type multinucleated giant cells were detected. The band of fibrosis adjacent to the lesion was moderate. Areas at a distance from the lesion revealed minimal granulocytic and no lymphocytic or plasma cellular infiltration. The infiltration with macrophages was minimal and no foreign body-type multinucleated giant cells were observed. The band of fibrosis was narrow in areas away from the wound. The barrier value of polymorphonuclear granulocytes was shown to be a minimal infiltration but without any lymphocytes or plasma cells. There was a mild infiltration with macrophages. No foreign body-type multinucleated giant cells were present. The barrier value of the control group revealed a narrow band of fibrosis (Fig. 1a). The results of the tissue reaction of the control group are given in detail in Table 3 and Fig. 1a.

#### Adept^®^

In the Adept^®^ group (*n* = 7) flat mesothelium-covered connective tissue with blood vessels and subjacent muscle was present with a minimal polymorphonuclear granulocytic infiltration and no presence of lymphocytes or plasma cells in the areas near the barrier (Fig. 4c). The infiltration with macrophages was mild (Fig. 4d) and no foreign body-type multinucleated giant cells were seen. Near the barrier, a moderate fibrous band was apparent. In areas far from the barrier, a mild polymorphonuclear granulocytic but no lymphocytic or plasma cellular infiltration was seen. The infiltration with macrophages was mild and no foreign body-type multinucleated giant cells were present in remote areas. The band of fibrosis was narrow in the areas at a distance from the barrier. The barrier value of the Adept^®^ group gave minimal amounts of polymorphonuclear granulocytes and no lymphocytes or plasma cells. The infiltration with macrophages was minimal and no foreign body-type multinucleated giant cells were detectable. The band of fibrosis was narrow (Fig. 1b). Table 3 and Fig. 1b provides a detailed description of the results of the Adept^®^ group.

#### Intercoat^®^

In areas near the barrier, the animals treated with Intercoat^®^ (*n* = 7) presented flat mesothelial cells on top of connective tissue with blood vessels with a minimal infiltration of polymorphonuclear granulocytes and no lymphocytes or plasma cells (Fig. 4e). The infiltration with macrophages was moderate to severe (Fig. 4f). No foreign body-type multinucleated giant cells were present and the band of fibrosis was broad adjacent to the barrier. Distant areas revealed no polymorphonuclear granulocytes and no lymphocytes/plasma cells. Infiltration with macrophages was moderate and notably less compared to the infiltration adjacent to the barrier. No foreign body-type multinucleated giant cells were seen and the band of fibrosis was narrow near the barrier. The barrier value of the Intercoat^®^ group exhibited mild amounts of polymorphonuclear granulocytes but no lymphocytes or plasma cells. The infiltration with macrophages was moderate to severe. No foreign body-type multinucleated giant cells were present. The band of fibrosis was of a moderate degree (Fig. 2a). Detailed information about the tissue response to Intercoat^®^ is given in (Table 4 and Fig. 2a).

#### Spraygel^®^

**Fig. 5:**
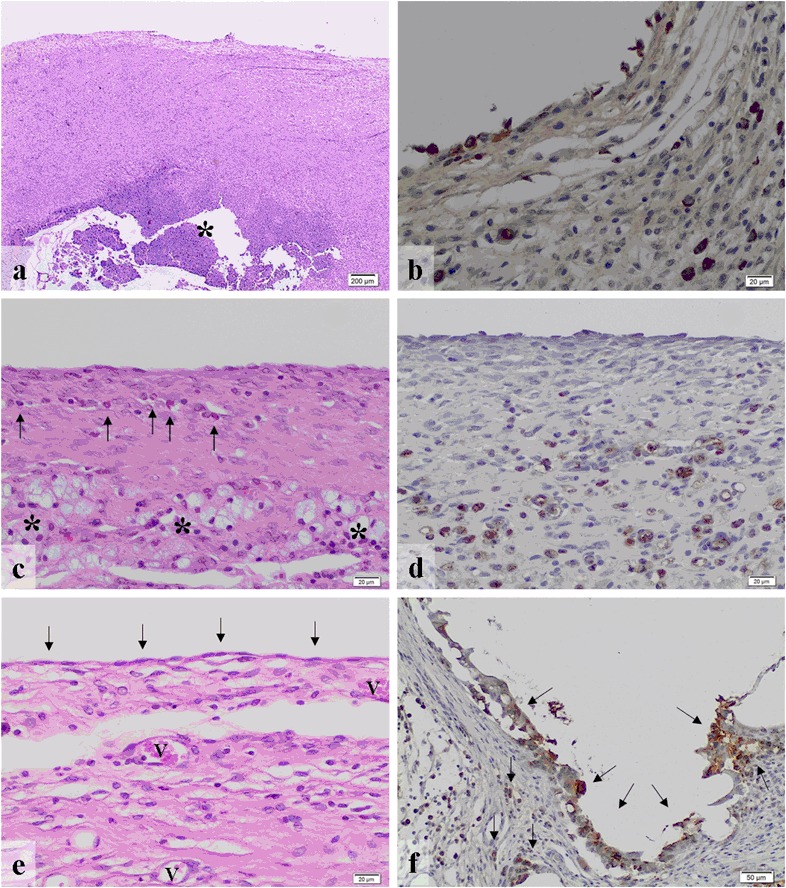
(**a**) In the Spraygel^®^ group submesothelial abscesses (*) were present (HE ×20) as well as (**b**) a moderate infiltration with macrophages (CD68 staining ×200). (**c**) Seprafilm^®^ revealed minimal amounts of granulocytes (*arrows*) and lymphocytes (*) (HE ×200) as well as (**d**) moderate infiltration with macrophages (CD68 staining, ×200). (**e**) In the SupraSeal^®^ group, a dense layer of flat mesothelial cells (*arrows*) covered connective tissue containing blood vessels (v) (HE ×200). (**f**) There was a high presence of macrophages (*arrows*) adjacent to the barrier (CD68 staining, ×100)

In the Spraygel^®^ group (*n* = 7) submesothelial abscess formation was detected with severe infiltration of polymorphonuclear granulocytes and cell detritus (Fig. 5a). In the absence of abscesses, the tissue often showed only few inflammatory cells. In the entire assessment a mild infiltration with polymorphonuclear granulocytes and no lymphocytes/plasma cells were observed in the areas near the barrier (Table 4). The infiltration with macrophages was moderate (Fig. 5b). No foreign body-type multinucleated giant cells were present near the barrier and the band of fibrosis was moderate. Areas at a distance from the barrier showed minimal amounts of polymorphonuclear granulocytes and no lymphocytes or plasma cells. The infiltration with macrophages was mild and no foreign body-type multinucleated giant cells were present in areas distant to the barrier. Furthermore, the band of fibrosis was narrow. The barrier value revealed a mild polymorphonuclear granulocytic infiltration and no lymphocytes or plasma cells. The infiltration with macrophages was moderate. No foreign body-type multinucleated giant cells and no fibrosis were present. Table 4 and Fig. 2b provides the results of the tissue response to Spraygel^®^ in detail.

#### Seprafilm^®^

In the animals treated with Seprafilm^®^ (*n* = 7) a mild infiltration with polymorphonuclear granulocytes and no lymphocytes or plasma cells were seen near the barrier (Fig. 5c). The infiltration with macrophages was moderate (Fig. 5d). No foreign body-type multinucleated giant cells were present and the band of fibrosis was moderate near the barrier. Areas at a distance from the barrier revealed neither polymorphonuclear granulocytes nor lymphocytes or plasma cells. The infiltration with macrophages was mild and no foreign body-type multinucleated giant cells were present. The barrier-remote areas exhibited a narrow band of fibrosis. The barrier value of the Seprafilm^®^ group presented a minimal polymorphonuclear granulocytic infiltration without any lymphocytes or plasma cells. The infiltration with macrophages was moderate and no foreign body-type multinucleated giant cells were detected. The band of fibrosis was narrow (Fig. 3a). The detailed results of the Seprafilm^®^ group are shown in Table 5 and Fig. 3a.

#### SupraSeal^®^

In the SupraSeal^®^ group (*n* = 7) a dense layer of flat mesothelial cells covered connective tissue containing blood vessels (Fig. 5e). The infiltration with polymorphonuclear granulocytes was moderate and lymphocytes or plasma cells were not present in areas near the barrier (Table 5). The infiltration with macrophages was moderate (Fig. 5f) and no foreign body-type multinucleated giant cells were present near the barrier. The band of fibrosis was narrow. In the areas away from the barrier, neither polymorphonuclear granulocytes nor lymphocytes or plasma cells were present. A minimal infiltration with macrophages was observed and no foreign body-type multinucleated giant cells were seen. The band of fibrosis was narrow in the remote areas. The barrier value of the SupraSeal^®^ group revealed a moderate polymorphonuclear granulocytic infiltration but without lymphocytes and plasma cells. The barrier value of macrophages presented a moderate to severe infiltration. No foreign body-type multinucleated giant cells no fibrosis was seen (Fig. 3b). Detailed information about the tissue response to SupraSeal^®^ is provided in Table 5 and Fig. 3b.

### Comparison of the different parameters of the tissue response

In the following, the tissue response to the barriers is directly compared with respect to the barrier value regarding inflammation, foreign body reaction, fibrosis and the infiltration of macrophages (Table 6).

**Table 6:** The tissue response to the barrier types according to [30]

Barrier	Inflammation		Foreign body reaction	Extent of fibrosis	CD68-positive macrophages
	Polymorphonuclear granulocytes	Lymphocytes/plasma cells			
Control	Minimal	None	None	Narrow band	Mild
Adept^®^	Minimal	None	None	Narrow band	Minimal
Intercoat^®^	Minimal	None	None	Moderate band	Moderate–severe
Spraygel^®^	Mild	None	None	No fibrosis	Moderate
Seprafilm^®^	Minimal	None	None	Narrow band	Moderate
SupraSeal^®^	Moderate	None	None	No fibrosis	Moderate–severe

#### Comparison of the inflammatory response

The granulocytic inflammation was lowest in the animals treated with Intercoat^®^ and Seprafilm^®^, both presenting a median of 1.1 cells per high power field. The control group (median: 1.8 cells per high power field) and the Adept^®^ group (median: 2.5 cells per high power field) followed. According to the quantity of cells, these four groups presented a minimal granulocytic inflammation. Spraygel^®^ (median: 7 cells per high power field) revealed a mild granulocytic inflammation. With a median of 21.5 cells per high power field, SupraSeal^®^ was associated with the strongest granulocytic reaction.

Lymphocytes or plasma cells were only seen sporadically in small numbers. Regarding the scoring system used in this study, there was no infiltration of lymphocytes or plasma cells in any of the analysed groups in this study. SupraSeal^®^ revealed none of these cells per high power field. Intercoat^®^ and Spraygel^®^ both presented a median of 0.1 cells per high power field and the control group as well as the Seprafilm^®^ group were both associated with a median of 0.2 cells per high power field. Adept^®^ followed with 0.3 cells per high power field.

Altogether, the inflammatory reaction was characterized by granulocytic infiltration with a negligible lymphocytic or plasma cellular reaction in all groups. Intercoat^®^, Seprafilm^®^, Adept^®^ and the control group revealed a minimal inflammatory response. The animals treated with Spraygel^®^ revealed mild inflammation, whilst the SupraSeal^®^ group presented a moderate inflammatory response.

#### Comparison of the foreign body reaction

No or regarding the score only minimal numbers of foreign body giant cells were present in any group. The barrier value of Intercoat^®^, Seprafilm^®^ and the control group revealed no foreign body-type multinucleated giant cells per high power field at the median. Spraygel^®^ followed, presenting 0.1 cells per high power field. Adept^®^ and SupraSeal^®^ both revealed a median of 0.5 cells per high power field in this study.

#### Comparison of the induced fibrosis

Seprafilm^®^, Adept^®^ and the control group revealed a narrow band of fibrosis. The Intercoat^®^ group presented a moderate band of fibrosis in this study. In animals treated with SupraSeal^®^ and Spraygel^®^ no fibrosis was present.

#### Comparison of the amount of macrophages

The presence of macrophages was lowest in the Adept^®^ group (median: 3.2 cells per high power field), which revealed a minimal response. The control group (median: 5.2 cells per high power field) followed and presented a mild reaction. The animals treated with Spraygel^®^ (median: 10.3 cells per high power field) and Seprafilm^®^ (median: 10.5 cells per high power field) were associated with a moderate response, whilst the Intercoat-^®^ (median: 28.8 cells per high power field) and SupraSeal^®^ group (median: 32.4 cells per high power field) revealed a moderate to severe reaction.

### Correlation between the tissue response and the amount of macrophages

The correlation analysis performed via the Pearson product-moment correlation coefficient revealed significant correlations between the foreign body reaction and the amount of macrophages in the Adept^®^ group (*r* > 0.6, *p* < 0.05), whereas a negative correlation between macrophage infiltration and the amount of fibrosis was found in the SupraSeal^®^ group (*r* < −0.6, *p* < 0.05). A correlation was present between the quantity of macrophages and the presence of granulocytes in the Intercoat^®^ group (*r* > 0.6, *p* < 0.1), whereas this trend was not seen in the evaluation of all samples, where no correlation was found (−0.6 < *r* < 0.6, *p* < 0.1). The control group as well as the Adept^®^, Spraygel^®^ and Seprafilm^®^ groups all showed no correlation between macrophage and granulocytic infiltration (−0.6 < *r* < 0.6). Regarding lymphocytes and plasma cells, a positive trend was seen in the relationship between the amount of these cells and the presence of macrophages in the SupraSeal^®^ group (*r* > 0.6, *p* < 0.1). Although not statistically significant, this result matched the findings in the Spraygel^®^ group (*r* > 0.6, *p* > 0.1), whereas other groups revealed no correlation between lymphocytic and macrophage infiltration (−0.6 < *r* < 0.6, *p* > 0.1). Concerning the foreign body reaction the correlation analysis performed via the Pearson product-moment correlation coefficient showed a significant positive correlation between the number of macrophages and the presence of foreign body giant cells in the Adept^®^ group (*r* > 0.6, *p* < 0.05). Interestingly, in the Seprafilm^®^ group a negative correlation trend was detected (*r* < −0.6, *p* < 0.1), whereas in the animals of the control group and those treated with Spraygel^®^ and SupraSeal^®^ no correlation was seen (−0.6 < *r* < 0.6, *p* > 0.1). A significant negative correlation between the infiltration of macrophages and the amount of fibrosis was seen in the SupraSeal^®^ group (*r* < −0.6, *p* < 0.05). In the other groups no correlation was detected (−0.6 < *r* < 0.6, *p* > 0.1). The results of the correlation analysis performed via the Pearson product-moment correlation coefficient are presented in Table 7.

**Table 7:** Results of the correlation analysis performed via Pearson product-moment correlation coefficient: Significant correlations were seen regarding the foreign body reaction and the amount of macrophages in the Adept^®^ group (*r* > 0.6, *p* < 0.05)

Barrier group		Granulocytes	Lymphocytes/plasma cells	Foreign body giant cells	Extent of fibrosis
Control group	Pearson-Corr.	−0.179	−0.390	0.040	0.165
*n* = 7	P	0.702	0.387	0.932	0.724
Adept®	Pearson-Corr.	0.123	0.228	0.839	0.300
*n* = 7	P	0.793	0.624	0.018	0.514
Intercoat^®^	Pearson-Corr.	0.727	0.209		0.246
*n* = 7	P	0.064	0.653		0.595
Spraygel^®^	Pearson-Corr.	−0.402	0.669	−0.311	−0.023
*n* = 7	P	0.371	0.101	0.497	0.961
Seprafilm^®^	Pearson-Corr.	−0.403	−0.217	−0.711	0.367
*n* = 7	P	0.370	0.640	0.073	0.418
SupraSeal^®^	Pearson-Corr.	0.491	0.712	0.324	−0.833
*n* = 7	P	0.263	0.073	0.478	0.020
All samples	Pearson-Corr.	0.264	−0.167	0.017	−0.101
*n* = 42	P	0.091	0.290	0.917	0.526

Also, a significant negative correlation between macrophage infiltration and the amount of fibrosis was found in the SupraSeal^®^ group (*r* < −0.6, *p* < 0.05). Statistical trends were seen between the amount of macrophages and the infiltration of granulocytes (positive correlation in the Intercoat^®^ group, *r* > 0.6, *p* < 0.1 and no correlation regarding all samples, −0.6 < *r* < 0.6, *p* < 0.1) as well as between the presence of macrophages and the lymphocytic infiltration in the SupraSeal^®^ group (*r* > 0.6, *p* < 0.1). Further, a trend to negative correlation between macrophages and the amount of foreign body giant cells was seen in the Seprafilm^®^ group (*r* < −0.6, *p* < 0.1). Although not all results are significant, mainly due to a low number of animals per group, the results are interesting since they show that there is no clear correlation between the amount of macrophages and the biocompatibility of barrier materials regarding inflammation, foreign body reaction or fibrosis

Altogether, significant correlations were seen between the foreign body reaction and the number of macrophages and a negative correlation was present between macrophage infiltration and the amount of fibrosis. Statistical trends were found between the amount of macrophages and granulocytes, with a positive correlation in the Intercoat^®^ group (*r* > 0.6, *p* < 0.1) but no correlation for total samples (−0.6 < *r* < 0.6, *p* < 0.1). Moreover, a trend was seen regarding the presence of macrophages and lymphocytic infiltration in the SupraSeal^®^ group (*r* > 0.6, *p* < 0.1), as well as a negative correlation between macrophages and the number of foreign body giant cells in the Seprafilm^®^ group (*r* < −0.6, *p* < 0.1). Not all results reached statistical significant, but overall it could be shown that there is no clear correlation between the quantity of macrophages and the tissue biocompatibility of barrier materials regarding inflammation, foreign body reaction or fibrosis.

## Discussion

Postoperative adhesion formation still represents a serious clinical problem [25] and the pathomechanisms of this condition are not yet completely understood. Furthermore, adhesion formation seems to be the consequence of the malfunction or imbalance of various mechanisms and factors occurring during or combined with peritoneal wounding [14, 16]. Macrophages play a crucial role in the modulation and regulation of inflammation and the immune response, tissue repair, the induction and formation of fibrosis as well as the elimination of pathogens [27]. From this point of view, these cells might also play a distinctive role in postoperative adhesion formation. In the present study, the tissue response to five commercially available adhesion barriers and a sham-operated control group was semi-quantitatively assessed with respect to inflammation, foreign body reaction, extent of fibrosis and the immigration of macrophages to evaluate a possible correlation between the tissue response and macrophage infiltration. As an important result, no correlation between the number of macrophages and any of the relevant parameters for tissue reaction could be detected, even if the total amount of macrophages differed within the various groups. In this context it was an interesting finding that in the untreated control group without any biomaterial implant a mild macrophage infiltration was seen, whereas in the animal group treated with Adept^®^ merely a minimal infiltration of macrophages was evident. In fact, there was a moderate infiltration in the Spraygel^®^ group and a moderate to severe infiltration in the animals treated with SupraSeal^®^. However, both latter groups showed no fibrosis. In contrast, the Intercoat^®^ group revealed a moderate to severe macrophage infiltration with a moderate band of fibrosis. However, in the control and the Adept^®^ group low counts of macrophages were seen combined with minor fibrosis. So, moderate macrophage infiltration was seen with both no and moderate extent of fibrosis. Also, low infiltration of macrophages was detected with minimal fibrosis (Table 6) [26]. These results are of special interest in view of the existing literature, since macrophages are thought to be the master regulators of fibrosis, which is controlled by several cytokines and signaling molecules, especially by TGF-β1 [31]. It still has to be clarified whether this regulation is based on the quantity of cells in the tissue or if it is mainly regulated at a molecular level given by the type and level of secreted cytokines, growth factors or signaling molecules. It is evident that a count of macrophages does not provide information about cell function. So in the present study, moderate macrophage infiltration was seen with both minimal and moderate inflammation (Table 6). Hence, inflammation was moderate in the SupraSeal^®^ group with a moderate to severe macrophage infiltration. By contrast, Intercoat^®^ and Seprafilm^®^ revealed a minimal inflammatory response with moderate and moderate to severe infiltration with macrophages, respectively. Both the Adept^®^ and the untreated control group showed minor values for overall inflammation and macrophage infiltration.

Macrophages were classically defined as phagocytic cells, but in recent experimental studies it has become apparent that they play a key role in the regulation of wound healing, inflammation and fibrosis [28, 32, 33]. According to their activity as important regulators, the view of these cells has changed completely, so that macrophages are no longer described as a homogenous population of cells but are divided into various subgroups according to their functions [32–34]. The ‘classically activated macrophages’, so called M1 macrophages, play an important role in host defence. These cells eliminate pathogens via phagocytosis and the production of O_2_ and N_2_ radicals [35]. Furthermore, they participate in the degradation of the extracellular matrix during inflammation [33] by secreting various enzymes, including collagenases, elastase and hyaluronidase [36]. The ‘alternatively activated macrophages,’ or M2 macrophages, are divided into at least three subpopulations. Each group of these has various functions, which are just beginning to be understood. In fact, some of these cells, the M2a subtype, appear to play a crucial role in wound healing and tissue remodelling by producing proteins of the extracellular matrix [37, 38]. Another subpopulation, the M2b macrophage, is believed to play a central role in the regulation and modulation of inflammatory immune responses and thereby limits tissue damage. The function of this macrophage type seems to be of a purely regulatory quality since these cells synthesize and secrete cytokines, growth factors and signalling molecules. In contrast to the other subpopulations of M2 macrophages, this type of macrophage does not produce extracellular matrix proteins by itself and hence does not actively participate in wound repair [39]. The third described phenotype of M2, the M2c macrophage, is crucially involved in immune suppression as well as in the modification, reorganization and degradation of the extracellular matrix. These cells could be of special interest with respect to postoperative adhesion formation since they are actively involved in the induction of fibrosis [37]. It is thought that, on the one hand, M2 macrophages produce cytokines and chemokines, which induce chemotaxis, proliferation and activation of fibroblasts into the lesion [40–43]. On the other hand, these cells are capable of producing components of the extracellular matrix, such as fibronectin [27]. Therefore, the functions and mechanisms of the various macrophage subpopulations might play an essential role in the formation of postoperative adhesions [26].

## Conclusion

In this study, no correlation was seen between the total infiltration of macrophages and the tissue response in terms of inflammation, foreign body reaction and fibrosis. The positive reaction for CD68 detects all types of macrophages without differentiating the various macrophage phenotypes. Based on these findings, it appears that the count of CD68-positive macrophages is no longer expedient for the evaluation of the tissue response or biocompatibility of material implants. As a consequence, since macrophages play decisive roles in the regulation of the immune response, wound repair and the host responses to biomaterial implants [33], this cell type and its role in tissue response to biomaterials should be further investigated also including its subtypes. For this purpose, markers have to be defined to precisely identify the various subgroups in the important animal model species as well as in humans. Moreover, with respect to peritoneal adhesions, in further studies the macrophage subpopulations should be assessed in postoperative adhesion formation with and without the treatment with barrier materials. These results should then be compared with the tissue response regarding inflammation, foreign body reaction and fibrosis to explore a possible correlation and thereby possibly open up new strategies for the therapy or prevention of postoperative adhesion formation.
